# Bridging Technology and Healthcare: A Bibliometric Review of Assistive Technologies in Hospital Environments

**DOI:** 10.3390/healthcare13233009

**Published:** 2025-11-21

**Authors:** Debopriyo Roy, Eleni Gkiolnta, George F. Fragulis

**Affiliations:** 1 Technical Communication Laboratory, Center for Language Research, Department of Computer Science & Engineering, The University of Aizu, Aizuwakamatsu 965-8580, Japan; droy@u-aizu.ac.jp; 2 Department of Educational & Social Policy, University of Macedonia, 54636 Thessaloniki, Greece; egkiolnta@uom.edu.gr; 3 Department of Electrical and Computer Engineering, University of Western Macedonia, 50100 Kozani, Greece

**Keywords:** assistive technologies (ATs), healthcare systems, patient profiling, bibliometric analysis, hospital applications

## Abstract

Today, healthcare systems face many challenges due to the increasing number of elderly people and the complex needs of patients with multiple diseases. Previous research has shown that assistive technologies (ATs), like wearable devices, mobile health (mHealth) apps, and smart monitoring systems, can help improve patient care and make healthcare services more efficient. However, many of these studies do not focus so much on hospitals and do not clearly show the effects on clinical outcomes. In this study, the authors conducted a bibliometric analysis using the Scopus database to determine how much research has been carried out on assistive technologies in hospitals, especially for patient profiling and treatment. The authors chose articles from the last 20 years using specific inclusion and exclusion criteria, and VOSviewer software (version 1.6.20) was used to study keywords, co-authorship, and citation networks to find research trends and missing areas. The results show that even if assistive technologies are growing fast, there are not many studies that focus on hospitals or on important outcomes like quality of care and treatment results. Most of the research is in computer science and engineering, and many keywords for hospital use are not common. This study discusses how assistive technologies can help change healthcare and also shows the current problems, like system integration, data privacy, cost, and whether users accept the technologies. The authors suggest that future research must look at personal solutions, international standards, and better cooperation between doctors, engineers, and policymakers.

## 1. Introduction

Recent research in computer science and healthcare informatics increasingly focuses on the global aging population and related challenges, particularly the importance of assistive technologies in hospital and home settings [1]. Because the number of people over 60 years old is rapidly growing, there appears to be an urgent need to transform healthcare services and develop far-reaching technologies to address complex health problems and prevalent diseases among older adults.

Assistive technology includes a variety of tools and devices designed to help people with disabilities. Assistive technologies encompass tools and devices that support individuals with disabilities in vision, hearing, mobility, communication, and daily activities, including screen readers, hearing aids, cognitive aids, and mobility devices [2]. Examples include screen readers for visual impairments, hearing aids for hearing loss, and speech-to-text systems that assist users with communication difficulties.

Modern assistive technologies—such as wearable sensors, mobile health (mHealth) apps, and home monitoring systems—enable real-time health monitoring and support independent living among older adults. These systems assist with real-time health data and help the elderly live independently. Current priorities include developing automated, AI-enabled, and remote-care systems to address resource limitations and enhance seniors’ quality of life. Ongoing efforts aim to develop accessible and user-friendly digital health tools for technologically experienced older adults. Recent studies have explored comprehensive care models, specific assistive tools, and integrated monitoring platforms for older adults, particularly those with dementia [3].

Relevant research studies on assistive technologies in hospital environments are fundamentally different from studies situated in home-based or rehabilitation contexts. Home and rehabilitation research are typically focused on personalized device usability, patient autonomy, and daily living support, while hospital-based research emphasizes system-level integration, interoperability, and optimization of clinical workflows. The hospital setting typically integrates additional layers of complexity, including regulatory compliance, data privacy, and alignment with institutional electronic health record (EHR) systems. Ref. [4] noted that most assistive technology studies emphasize user experience and quality-of-life enhancement in domestic or rehabilitative settings. However, integration in hospital care demands a stronger focus on infrastructure and interoperability. Similarly, ref. [5] highlighted that while home-based technologies are evaluated for accessibility and usability, hospital-based applications necessitate standardization in terms of safety, reliability, and compliance within multidisciplinary clinical teams. Consequently, hospital-focused research examines assistive technologies integral to broader healthcare ecosystems, whereas home-based studies are more inclined toward individual functionality and autonomy.

The evolution of assistive technology research indicates a progressive integration of user-centered design, data-driven innovation, and policy-informed frameworks. Earlier systematic reviews by [4,6] researched the importance of evaluating functionality, adoption feasibility, and user perceptions, highlighting behavioral and institutional factors that shape technology uptake. This foundation allowed [5] to emphasize hospital-based situations that include equitable access and international standards, linking technical innovation to governance and policy. Concurrently, studies like [7,8] expanded the field toward data-centric and digitally integrated ecosystems, while [9] advanced personalized device development through rapid prototyping. Other research focused on cutting-edge applications in clinical and rehabilitative contexts, including hybrid brain–computer interfaces [7,10] and wearable AR systems [11], highlighting enhanced patient outcomes and procedural support. Telehealth and continuity-of-care models [12] represent another dimension that underscores the practical and economic benefits of assistive systems. Finally, ethical and governance considerations [13] have guided interdisciplinary research toward responsible and patient-centered implementation, collectively shaping the trajectory of assistive technology research.

In hospital settings, wearable and Internet of Things (IoT) technologies enable rapid and accurate monitoring of elderly patients. These devices generate diverse physiological and behavioral data, which, when combined with patient histories, require advanced analytical processing. Technologies like Big Data Analytics, cloud computing, machine learning, cognitive computing, and blockchain combine and coordinate to manage the data. These technologies promote continuous monitoring and better patient care plans. However, collecting and sending health data can cause big problems regarding privacy and security. These risks are heightened when older adults interact directly with technology. Therefore, digital health devices should be tailored to the physical and cognitive needs of older users to ensure both utility and safety [7].

Research by several authors [14,15,16,17,18] show many assistive technologies that help with seniors’ care. These support accessibility, safety, social life, and prevent injuries for seniors with physical or mental problems. Important technologies are as follows:

1. Ambient Assisted Living (AAL) systems that range from single devices to whole home systems to help seniors with daily life and health.
2. Wearable devices like smartwatches, smart fabrics, and patches for vital signs monitoring, emergency detection, and remote health support, for example, smart contact lenses and graphene patches for diabetes.
3. Mobile health (mHealth) uses phones and wireless networks for health services. It includes the following:
  ○ Self-healthcare management: the elderly monitor themselves using body sensors and mobile devices.
  ○ Assisted healthcare: carers share data and help in emergencies.
  ○ Supervised healthcare: doctors and caregivers monitor data all the time to manage care remotely.

These technologies aim to enhance quality of life, independence, and health outcomes among older adults, though challenges persist regarding user acceptance, system integration, and power efficiency.

The World Health Organization [19], in a global report about assistive technologies, forwarded 10 recommendations to improve access and move toward universal coverage. Many of them are important for hospital management to create a good environment for assistive technologies, and they include the following:

1. Improve access in all development areas.
2. Make assistive products safe, effective, and affordable.
3. Grow and improve workforce skills.
4. Include users and families actively.
5. Increase public knowledge and fight stigma.
6. Invest in data and policies based on evidence.
7. Invest in research, innovation, and good environments.
8. Develop and support enabling environments.
9. Use assistive technology in humanitarian aid.
10. Give technical and financial help through international cooperation.

This study does not assess the implementation of WHO recommendations in hospital-based assistive technology research; however, this represents an important area for future investigation. This bibliometric study provides a comprehensive overview of research trends in the Scopus database on assistive technologies for patient profiling and treatment in hospital settings.

Study Objective and Aim:

Objective: To analyze existing research on the use of assistive technologies in hospitals, with a focus on enhancing patient profiling, treatment efficiency, and healthcare outcomes.

The key contributions of this paper include the following:

1. Providing a comprehensive overview of global research trends on assistive technologies in hospital settings.
2. Highlighting predominant research themes and identifying underexplored areas related to patient profiling and treatment.
3. Discussing challenges and opportunities for integrating assistive technologies into clinical practice.
4. Suggesting directions for future research to improve healthcare outcomes for the elderly.

This study specifically aims to achieve the following:

1. Identify underrepresented hospital domains in assistive technology research by examining the weak linkage between technical innovation and hospital-based clinical application.
2. Map the thematic evolution of assistive technology research over the past two decades to trace how focus areas have shifted from device development to issues of integration, ethics, and patient-centered implementation.
3. To examine patterns of collaboration and disciplinary imbalance, particularly the dominance of computer science over medical research outputs, to reveal structural dynamics that may influence real-world adoption.

The primary contribution of the study lies in evaluating the translational gap between technological innovation and hospital integration—an aspect not explicitly addressed in previous bibliometric reviews of telemedicine, digital health, or wearable technologies. This framing enhances interpretability by positioning the study as a diagnostic mapping of thematic and disciplinary convergence rather than a general survey of publication trends.

In summary, the present study aims to provide a comprehensive bibliometric overview of assistive technology research within hospital settings, mapping its global trends, thematic structures, and collaboration patterns over the past two decades. It is important to note that while this study quantifies research activity and thematic development, it does not directly evaluate clinical or patient-level outcomes. Instead, the bibliometric findings serve to highlight where outcome-oriented studies are concentrated and where significant gaps remain, thereby offering a strategic foundation for future systematic reviews or meta-analyses that may investigate the clinical effectiveness, usability, and patient impact of assistive technologies in healthcare environments.

Research Questions:

The fundamental question guiding this study is: What is the global extent and focus of research on the effectiveness of assistive technologies in hospital and non-hospital settings for patient profiling and treatment, and how are these technologies applied in healthcare?

Specifically, this study addresses the following research questions:

(1) What are the global publication trends, disciplinary distributions, and collaboration patterns in assistive technology research within hospital environments from the past two decades?
(2) How do the thematic clusters and keyword co-occurrence networks reveal the conceptual evolution and interdisciplinary nature of this research field?
(3) In what ways does hospital-based assistive technology research differ from home-based or rehabilitation-oriented studies, particularly in its focus, institutional involvement, and integration challenges?
(4) Finally, what ethical, policy, and organizational barriers and enablers emerge from the existing literature, and how do these insights identify critical research gaps that can inform future outcome-oriented or meta-analytic investigations?

Comparison with prior bibliometric studies in telemedicine, smart healthcare, and wearable technologies:

Previous bibliometric studies in related domains, such as telemedicine, smart healthcare, and wearable technologies, have primarily mapped technology diffusion and service innovations across home, community, and clinical contexts. For instance, ref. [20] conducted a large-scale bibliometric analysis of telemedicine during the COVID-19 pandemic and found that publication growth was driven primarily by the global shift toward remote care models and digital consultations. Similarly, ref. [21] examined the evolution of smart healthcare and highlighted dominant research hotspots in artificial intelligence (AI), Internet of Things (IoT), and big data analytics, without focusing on specific clinical environments. Studies on wearable sensors, such as those by [22,23], have demonstrated technology in health monitoring and fitness tracking but treated hospitals as only one among many potential sites of application. When compared to these broader approaches, the present bibliometric study is distinct in its exclusive focus on assistive technologies within hospital settings, particularly those addressing patient profiling and treatment. By limiting the analysis to Scopus-indexed works between 2014 and 2024, this study exposes patterns that other reviews overlook—specifically, the weak co-occurrence between the keywords “assistive technology” and “hospitals,” indicating a significant gap between technical innovation and clinical implementation within hospital settings.

Unlike earlier bibliometric mappings that primarily identified emerging technologies, this analysis combines co-word clustering and top-cited paper interpretation to explore why such gaps persist. The results reveal two parallel but uneven trajectories: one driven by engineering and computer science innovations, such as AI-based rehabilitation and wearable systems, and another still developing in clinical integration, interoperability, and policy frameworks. While previous telemedicine reviews emphasize adoption trends and access equity during the pandemic era, the present study advances the discussion by connecting bibliometric structures to gaps in policy actions, such as integration challenges, data privacy, interoperability, and cost-effectiveness. This analytical depth positions the study as both a descriptive mapping of research activity and a framework that could potentially diagnose where collaboration among technologists, clinicians, and policymakers must strengthen to achieve hospital-ready assistive technology ecosystems.

The remainder of this paper is organized as follows. Section 2 describes the bibliometric methodology and data collection process. Section 3 presents the analysis results, including research trends and gaps. Finally, Section 4 discusses the implications of these findings and proposes directions for future research.

## 2. Methods

**Figure 1:**
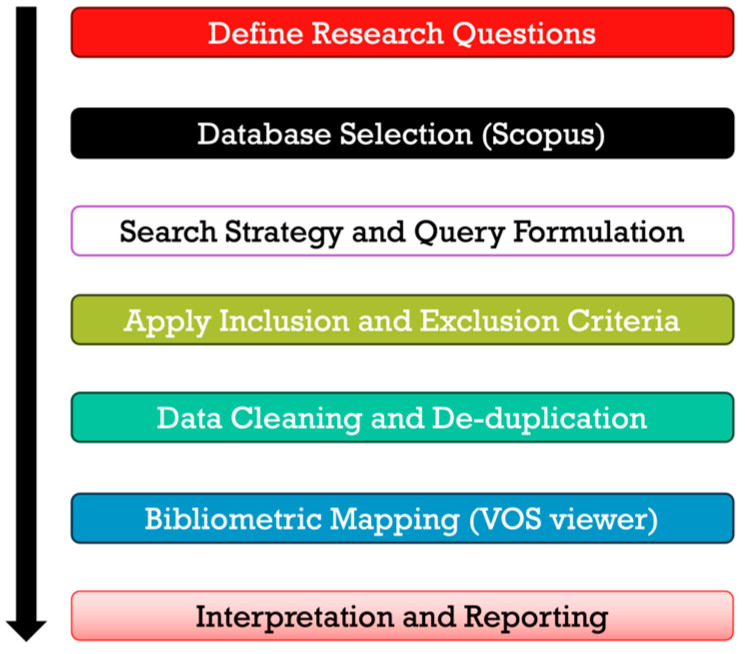
Framework for the bibliometric analysis conducted.

Only the Scopus database was utilized for this study. Scopus is a comprehensive abstract and citation database that offers extensive bibliometric information on academic publications, including citation counts, h-index scores, collaboration indicators, and other performance metrics. It is widely recognized for tracking global research productivity and emerging trends [24]. Figure 1 outlines the workflow of the analysis adopted for the Scopus search process, while Table 1 and Table 2 present the inclusion and exclusion criteria applied during keyword selection and dataset refinement.

Database Selection and Search Strategy:

The bibliometric dataset was extracted exclusively from the Scopus database because of its extensive interdisciplinary coverage and standardized indexing in computer science, engineering, and health-related disciplines. The search was conducted in April 2024, covering a 10-year period (2014–2024), to capture the evolution of assistive technology research and its integration into hospital environments.

The exact search string and Boolean logic used were as follows:
(TITLE-ABS-KEY (“assistive technology” OR “rehabilitation technology” OR “therapeutic device*” OR “assistive device*” OR “supportive technology”) AND TITLE-ABS-KEY (“hospital *” OR “clinical environment” OR “inpatient” OR “healthcare facility*” OR “medical center” OR “acute care”)) AND (LIMIT-TO (LANGUAGE, “English”)) AND (EXCLUDE (DOCTYPE, “Note”)) AND (EXCLUDE (SRCTYPE, “Book”))

This query retrieved a total of 1274 documents, including journal articles, conference proceedings, and reviews.

**Figure 2:**
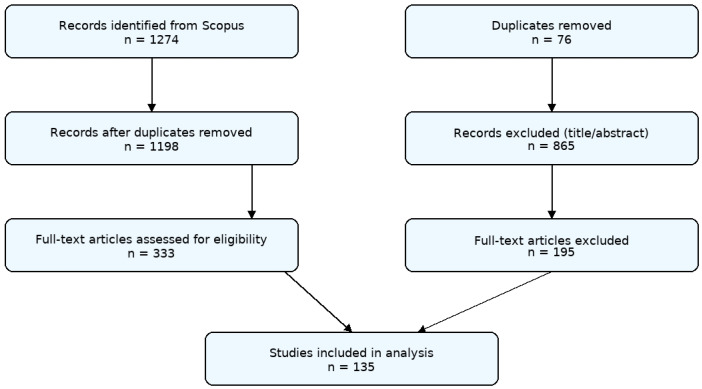
PRISMA framework for bibliometric search.

The PRISMA 2020 flow diagram (Figure 2) summarizes the data identification and screening procedures used in this bibliometric study. An initial 1274 records were retrieved from the Scopus database using iterative keyword combinations related to “assistive technology,” “hospital,” “rehabilitation,” and “treatment,” covering the period from 2014 to 2024. After removing 76 duplicate entries, 1198 records remained for title and abstract screening. Of these, 865 records were excluded due to irrelevance, non-English language, or incomplete bibliographic information. The remaining 333 full-text articles were then assessed for eligibility based on their relevance to research studies on hospital-based assistive technology. Following this step, 195 papers were excluded for reasons such as focus on homecare or community settings, incomplete metadata, or lack of hospital context. Finally, 135 records met the inclusion criteria and were retained for the bibliometric and network analyses conducted using VOSviewer. This process ensured methodological transparency, reproducibility, and adherence to PRISMA 2020 guidelines adapted for bibliometric studies [25].

Data Cleaning, Deduplication, and Validation:

The bibliographic data were exported in CSV format containing metadata fields such as title, author, affiliation, abstract, keywords, source, and citation count. The dataset underwent multi-stage cleaning using Microsoft Excel to ensure accuracy and consistency, as follows:

1. Duplicate removal: Records were matched and removed based on DOI, title, and author combinations.
2. Content screening: Editorials, book reviews, conference introductions, and notes were excluded.
3. Keyword verification: Entries missing author or indexed keywords were removed to maintain co-occurrence reliability.

To validate consistency, a random sample of 50 entries was cross-checked with Scopus for accuracy of metadata and citation data.

Bibliometric and Visualization Analysis Using VOSviewer:

Bibliometric mapping and visualization were conducted with VOSviewer version 1.6.20. Analyses included keyword co-occurrence, co-authorship, and country collaboration networks. The specific VOSviewer settings were as follows:

- Analysis Type: Co-occurrence (author keywords + indexed keywords).
- Counting Method: Full counting.
- Minimum Occurrences Threshold: 5.
- Normalization Method: Association strength.
- Visualization Modes: Network and density visualization.
- Clustering Resolution: 1.00 (default).
- Minimum Cluster Size: 10.
- Attraction/Repulsion Parameters: 2 and −1 (default).

The study merged similar terms (e.g., “assistive technology” and “assistive technologies”) and removed generic or non-descriptive words (e.g., “system,” “study,” “health”). Co-authorship mapping used thresholds of ≥5 documents per author and ≥10 documents per country.

A string of exact keywords and search phrases was used to find relevant studies in the Scopus database that were about the “effectiveness of assistive technologies in hospitals for patient profiling and treatment.” Here is a comprehensive search strategy as outlined in Table 3 and Table 4:

Keywords and Phrases:

**Table 3:** Keywords and phrases.

General Terms:	Patient Profiling:	Treatment:	Outcome and Impact
● “Assistive technologies” ● “Hospital” ● “Effectiveness”	● “Patient profiling” ● “Diagnostic tools” ● “Patient monitoring systems”	● “Treatment” ● “Rehabilitation devices” ● “Therapeutic devices”	● “Patient outcomes” ● “Clinical outcomes” ● “Healthcare efficiency” ● “Clinical workflow” ● “Treatment efficacy” ● “Patient care”

Search Strings:

Table 4 highlighted the search strings adopted for the study.

**Table 4:** Search strings.

Text	Figure
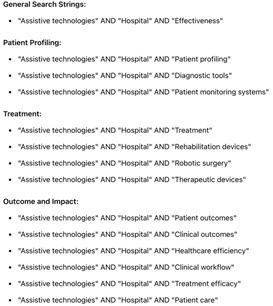	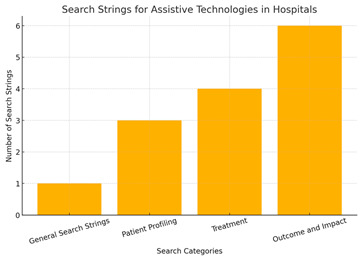

Bibliometric Review Methodology:

To strengthen the methodological rigor of this study, the bibliometric framework was grounded in established theoretical and analytical models widely recognized in scientometric research. The overall workflow and analytical structure followed the Bibliometrix framework proposed by [26], which provides a systematic approach for science mapping and performance analysis using R-based tools. The general procedures for dataset validation, indicator selection, and research field visualization adhere to the standardized bibliometric guidelines outlined by [25], ensuring transparency, replicability, and methodological consistency. For visualization and network construction, this study employs the VOSviewer software, whose clustering algorithms and association-strength normalization are theoretically based on the work of [27]. Complementary insights from [28,29] further inform the interpretive framework for analyzing bibliometric networks, particularly co-citation and thematic evolution. Collectively, these methodological foundations ensure that the present analysis aligns with accepted international standards in bibliometric research.

In addition, all references have been cross verified for bibliographic accuracy, and formatting has been standardized to include complete details such as journal titles, publication years, and DOIs for consistency across the manuscript.

Study Scope:

Additional methodological considerations were excluded due to the defined scope of the study:

1. Quality Assessment.
  ○ Exclude studies with poor methodological quality (e.g., low sample size, lack of control groups, high risk of bias) after quality assessment using tools like the Cochrane Risk of Bias tool or similar.
2. Relevance.
  ○ Exclude studies that do not directly evaluate the effectiveness of assistive technologies for patient profiling and treatment, even if the title and abstract make them seem relevant at first.

By adhering to these criteria, the bibliometric analysis focused on high-quality, relevant research, providing complete and correct information about how well assistive technologies in hospitals help with patient profiling and treatment.

This study was designed as a descriptive bibliometric mapping rather than a quantitative scientometric model. The principal aim was to visualize the distribution, thematic structure, and collaboration patterns in assistive technology research within hospital settings—not to test hypotheses or model temporal causality. Statistical techniques such as regression-based trend analysis, citation trajectory modeling, or thematic evolution tests require homogeneity of data and time-series completeness across multiple databases, which are beyond the defined scope of this study.

Given that the primary research question centered on the global extent and thematic distribution of research, the use of descriptive indicators (publication volume, citation count, co-occurrence density, and network clusters) was both appropriate and methodologically sufficient. This approach aligns with the exploratory nature of bibliometric mapping recommended by [26,27], where the goal is to identify structural patterns rather than perform statistical inference. Future studies may extend this work through scientometric modeling—for instance, analyzing growth trajectories or thematic evolution—but this article intentionally remains within the descriptive and diagnostic scope to establish a baseline understanding of how assistive technology research has evolved across disciplines and regions.

## 3. Results

The figures below illustrate how focusing on specific keyword searches resulted in a reduction in the number of research articles retrieved from the Scopus database.

**Figure 3:**
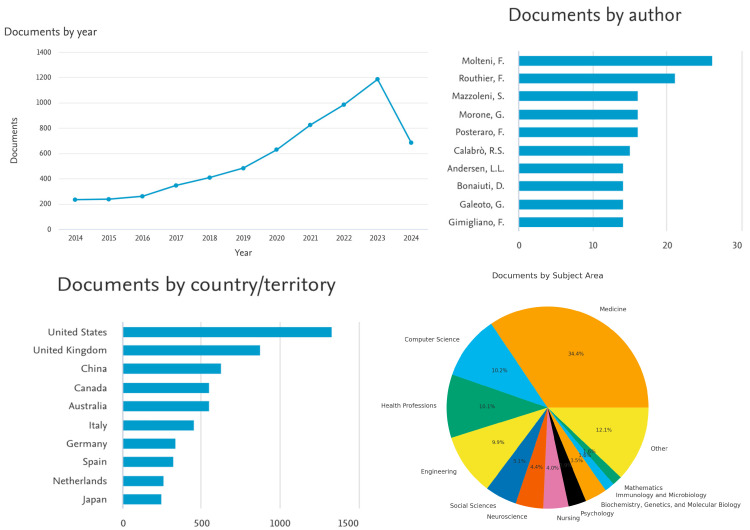
Key Scopus results for 6282 documents between 2014 and 2024.

More General (Figure 3):

Assistive + technology + hospital + effectiveness (2014–2024).

Results returned: 6282 documents.

**Figure 4:**
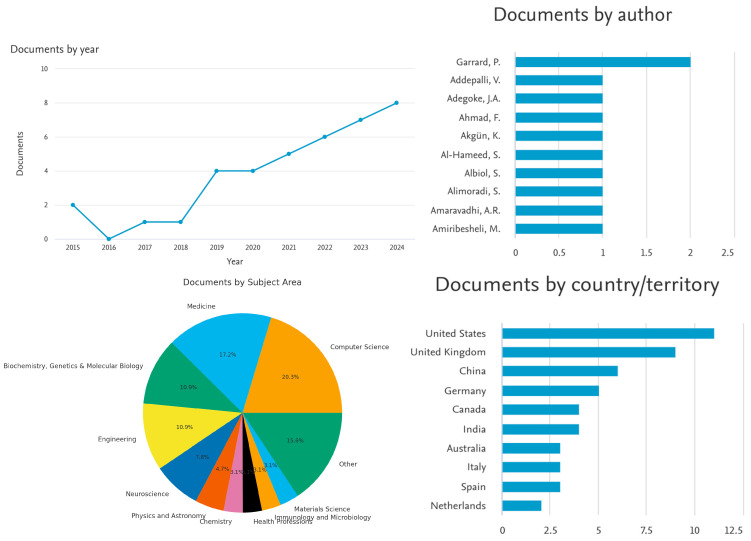
Key Scopus results for 38 documents between 2014 and 2024.

More Specific: Search for Patient Profiling (Figure 4):

Assistive technology + patient profiling + diagnostic tools + patient monitoring systems.

Keywords limited to: Machine learning, AI, diagnosis, procedures, algorithms, IoT, health care.

Results returned: 38 documents (2014–2024)

When limited to computer science and medicine, only 23 results were returned. With the above figures, we see an upshot in the number of publications on this topic starting in 2020. Not surprisingly, most of the publications are in the fields of computer science and medicine.

More Specific: For Outcome and Impact:

We conducted a different type of search to investigate keywords that had more impact in identifying articles that focused on outcomes and implications of assistive technologies.

An interesting observation is that when the Scopus database is searched with only “assistive technologies,” 10,084 articles are returned. Also, when the keywords in Table 4 are searched with an “AND” in between, many more search results are often returned for scrutiny. This indicates that this is still a new field of search, at least as registered in the Scopus database, and not many articles have these obvious keywords joined together, at least in the title or in the article content. Results also indicate that most of the articles are found in the fields of medicine, computer science, and engineering, led by publications in the USA. With only “assistive technologies” being searched in the Scopus database, the majority of the articles are found in computer science.

Table 5 goes on to show that research in assistive technology has more often been conducted without any reference to hospitals, patients, clinical outcomes, and healthcare efficiency. Only 520 articles were traced with “assistive technologies” and “hospitals” as keywords/phrases, and 9737 citations were recorded for these 520 articles.

To broaden the coverage of this study, additional searches were carried out in the Scopus database using alternative terms that could describe *assistive technologies in hospitals*. These terms were included to capture studies that might have used slightly different expressions for similar concepts. Table 6 presents the outcome of these searches. A notable pattern emerged: keywords that specify the *type* or *stage* of intervention—such as *rehabilitation technology* and *therapeutic technology*—returned more articles. In contrast, broader or less precise phrases like *healthcare assistive technology* and *health support technology* yielded very few results, while terms such as *assistive medical*, *clinical assistive technology*, and *mobility aids in healthcare* produced none.

Although these alternative terms are common in healthcare discussions, they do not appear to function as effective search identifiers in academic databases. It seems that in published literature, narrower and more technically defined keywords have greater visibility than general descriptors.

While Scopus served as the main source of bibliometric data, VOSviewer was used to interpret and visualize the findings through clustering and mapping techniques. At the exploratory stage, many combinations of terms were tested to determine the best coverage. For the final visualization and analysis, the search was narrowed to “assistive technology” AND “hospitals”, covering the years 2014–2024 and focusing on articles, conference papers, and book chapters in the fields of *medicine*, *health professions*, *computer science*, and *engineering*.

Using these refined criteria, 245 documents were identified and included for mapping and visualization. Additional Scopus searches also showed that most publications discussed *assistive technology* independently, with around 467 records combining *assistive technology* and *treatment*, and very few addressing *patient profiling*. To capture both clinical and broader healthcare perspectives, this study therefore included articles categorized under “assistive technology in hospitals” and “assistive technology for treatment.” Table 7 shows very specific search criteria that are perfect alternatives to “assistive technologies in hospital settings”.

Results from VOSViewer:

Several types of analysis have been conducted using VOSViewer. We have also conducted searches from the last 20 years (2004–2024) using the same kinds of keywords and inclusion/exclusion criteria.

Co-authorship Analysis:

A bibliometric network shows the connections between researchers, research groups, and countries based on how many journals they have co-authored together. This is carried out using co-authorship analysis.

**Figure 5:**
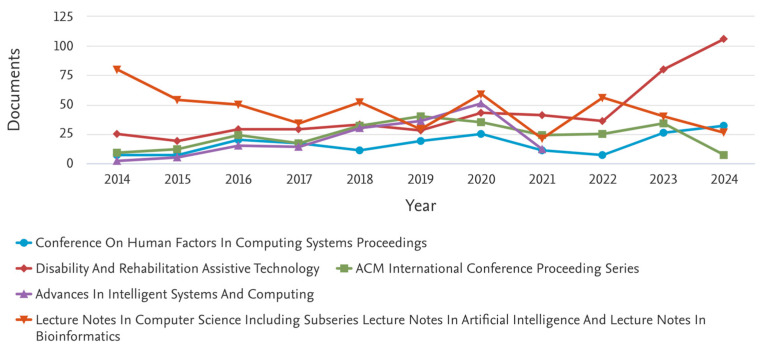
Documents Per Year by Source (10,084 articles).

**Figure 6:**
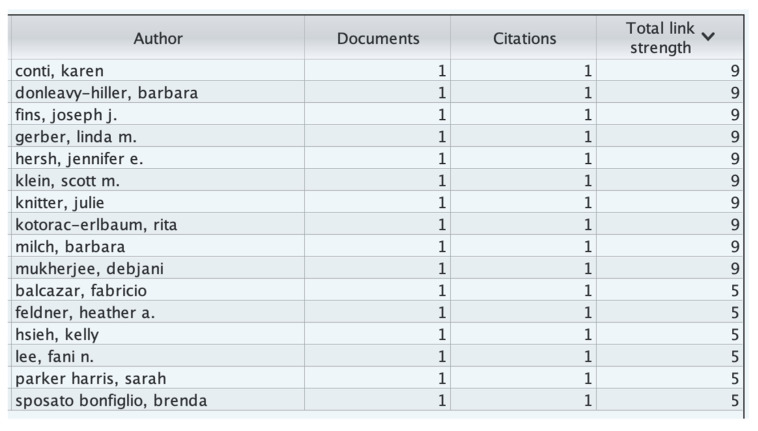
The total link strength for authors with a minimum of 1 citation has been shown.

**Figure 7:**
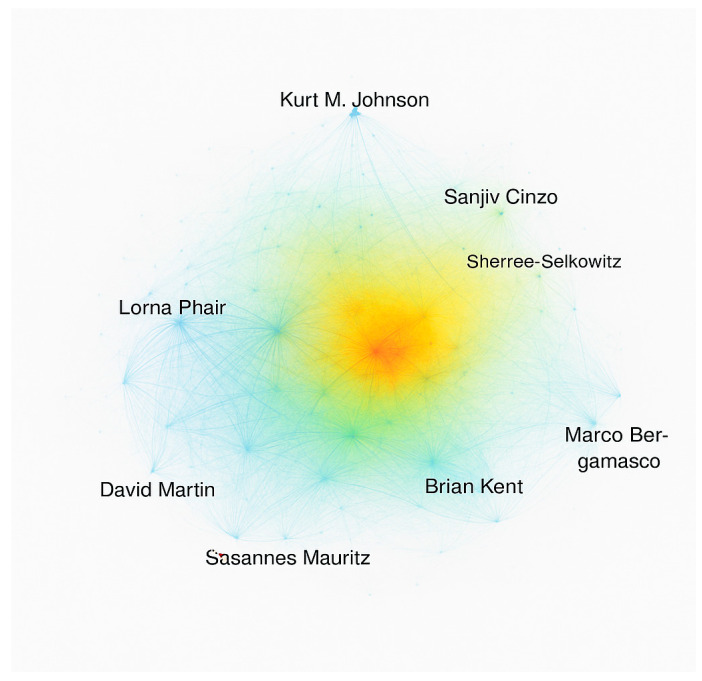
Density visualization of co-authorship networks in assistive technology research (2004–2024)—based on VOSviewer.

In our analysis based on Figure 5, Figure 6 and Figure 7, 16 authors satisfied the criteria. For each of the 16 authors, the total strength of the co-authorship links with other authors has been calculated. The authors with the greatest total link strength have been calculated, and 6 out of the 16 authors in the network are not connected. The largest set of connected items consists of 10 authors.

Figure 7 presents a density visualization map generated through co-authorship analysis, where each node represents an author and the color intensity reflects the level of collaboration activity. Brighter colors (yellow to red) indicate regions with higher collaboration density, showing authors who frequently co-publish or act as central connectors within the research network. Cooler colors (blue to green) represent less interconnected authors. The map includes the most active contributors (e.g., Brian Kent, Susanne Mauritz, Lorna Phair, and Marco Bergamasco), each linked through an average of nine co-authorship relationships. The visualization illustrates how collaborative clusters form around key researchers, highlighting the central hubs of knowledge production and interdisciplinary teamwork in assistive technology research within hospital environments.

**Figure 8:**
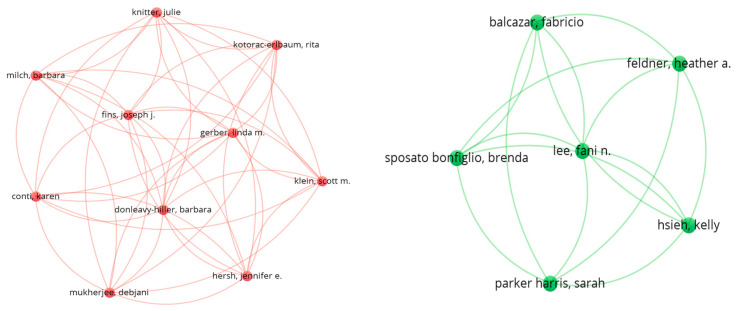
The red network has 9 links, while the green network has 5 links.

The red and green network visualization (Figure 8 depicts the co-authorship relationships among researchers publishing on assistive technology in hospital environments between 2004 and 2024. Each node represents an individual author, while the links (lines) between nodes indicate collaborative relationships formed through co-authored publications. The color-coded clusters reflect distinct collaboration communities, with each cluster grouping authors who frequently publish together or share overlapping research themes.

In this network, the red cluster represents a core group of authors primarily engaged in studies on *rehabilitation technologies*, *therapeutic devices*, *and patient-support systems*, often collaborating across clinical and engineering disciplines. The green cluster, on the other hand, includes authors focusing on *digital healthcare systems*, *artificial intelligence applications*, *and assistive technology integration in hospital workflows*. The size of each node corresponds to the author’s publication frequency or citation strength, while the thickness of the connecting lines indicates the strength of collaboration.

Overall, this figure illustrates the structure and intensity of research collaboration in the field, revealing that assistive technology research in hospital contexts is organized into a few highly connected author clusters with limited cross-linkages between them, highlighting the opportunity for greater interdisciplinary collaboration between clinical researchers and technology developers.

Co-occurrence with all keywords analysis:

Keyword co-occurrence analysis (co-word analysis) is conducted in VOSviewer to visualize the relationships of keywords or topics to one another. Co-word analysis is a text-mining technique that analyzes the “co-occurrence” of pairs of keywords in the review documents [30]. For this analysis, we set the minimum number of occurrences of a keyword to three. Of the 191 keywords, 12 met the threshold. For each of the 12 keywords, Figure 8 shows the total strength of the co-occurrence links with other keywords. The keywords with the greatest total link strength have been selected. A link means a co-occurrence connection between two keywords. According to the VOSviewer manual, each link has a strength, represented by a positive numerical value. The total link strength indicates the number of publications in which two keywords occur together. An interesting observation in Table 8 is that assistive technology has a link strength of 11, with a total link strength of 32. No other words or technology (e.g., IoT, machine learning, screen readers, hearing aids, speech-to-text software, adaptive keywords, etc.) directly linked to assistive technology appear in the list except for self-help devices.

Citation by Authors Analysis:

For this analysis, we set the minimum number of documents for an author to 1 and the minimum number of citations of an author to 1. Of the 59 authors, 16 meet the threshold. For each of the 16 authors, the total strength of the citation link with other authors has been calculated. The authors with the greatest total link strength have been selected.

The total link strength of an author is the sum of all co-authorship links they have with other authors in the dataset. A higher total link strength indicates a more collaborative or central researcher within the network, while a value of 0 means that the author’s publications are independent or not connected to others in the dataset through shared publications.

**Figure 9:**
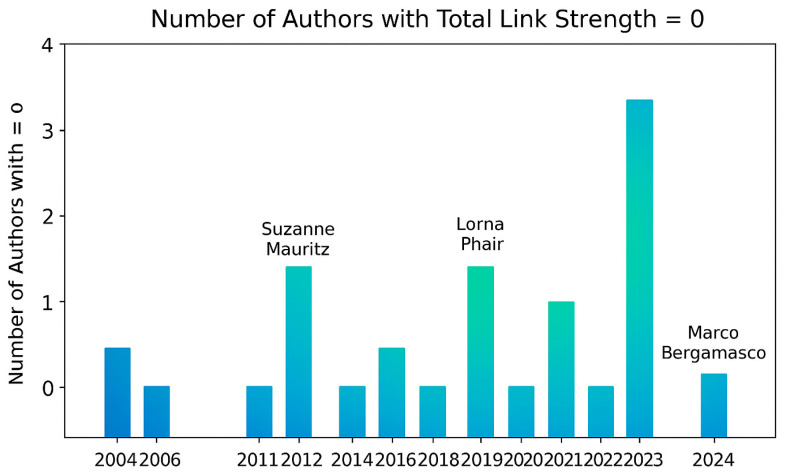
Number of authors with total link strength = 0.

Figure 9, titled “*Number of authors with total link strength = 0,*” visualizes authors who appear in the bibliometric dataset but have no recorded co-authorship links—that is, they conducted their research independently.

- The bars represent the number of such authors across different years (2004–2024).
- The color gradient (blue → green → yellow → red) reflects temporal or density-based intensity in line with VOSviewer’s visual logic.
- The labeled authors (e.g., Suzanne Mauritz, Lorna Phair, Marco Bergamasco) highlight key contributors who have published individually but not collaborated extensively within the Scopus-indexed assistive technology research community. This visualization helps identify isolated researchers or institutions and reveals potential collaboration gaps in the field.

**Figure 10:**
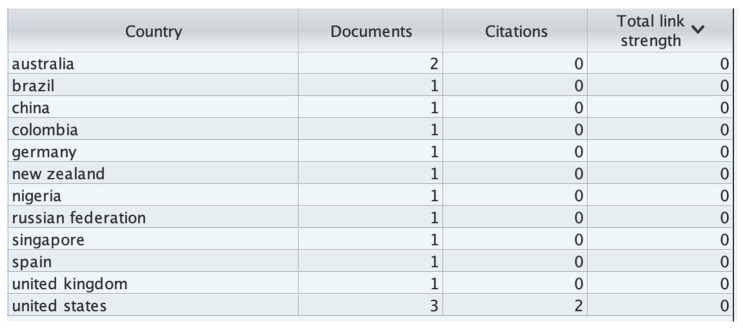
Except for the USA, no other country showed any citations.

In Figure 10, for each of the 12 countries, the total strength of the citation links with other countries has been calculated.

Bibliometric Coupling by Authors:

Bibliometric coupling by authors in VOSviewer is a method used to analyze and visualize the relationships between authors based on the number of references they share in their published works. When two authors cite the same documents in their publications, they are said to be bibliometrically coupled. By leveraging bibliometric coupling, researchers and analysts can gain insights into the collaborative landscape of a research field, identify key contributors, and understand the evolution of research topics over time.

**Figure 11:**
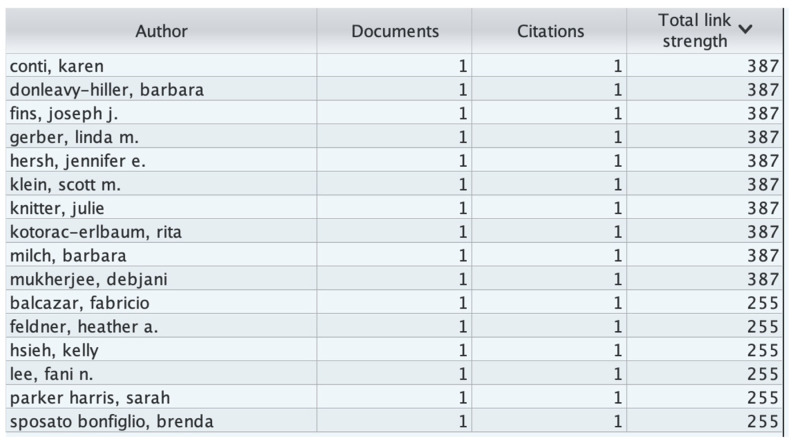
Total link strength for authors in the context of bibliometric analysis.

Figure 11 shows the number of citations by author and the total link strength. In the context of bibliometric coupling by authors in VOSviewer, the total link strength is a measure that quantifies the overall strength of an author’s connections to other authors within the network. It reflects the cumulative strength of all bibliometric coupling links that an author has with other authors in the network.

**Figure 12:**
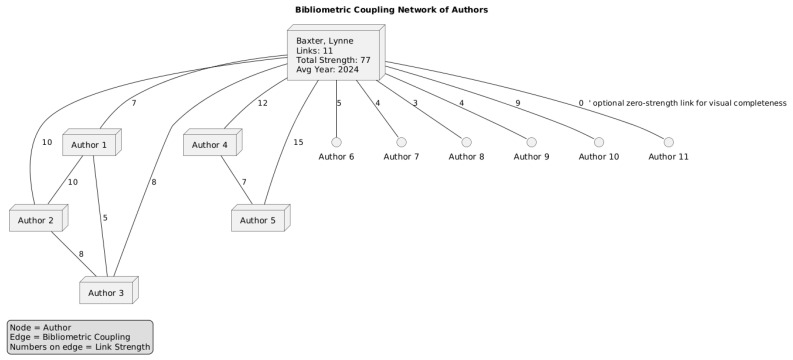
Bibliometric coupling network of authors.

For Figure 12, we set the number of citations of an author to 0, and 59 authors met the threshold. For each of the 59 authors, the total strength of the bibliometric coupling links with other authors was calculated. The authors with the greatest total link strength were selected. The analysis revealed that only 12 out of the 59 items in the network had connections. Baxter, Lynne (the isolated cluster on the right) has 11 links, with a total link strength of 77 and an average publication year of 2024.

Note: *The citation count of the author (total citations they have received) is independent of the link strength. Setting an author’s citation count to 0 does not automatically make their link strength 0. They could still have strong links if their publications cite many references that overlap with other authors.*

**Figure 13:**
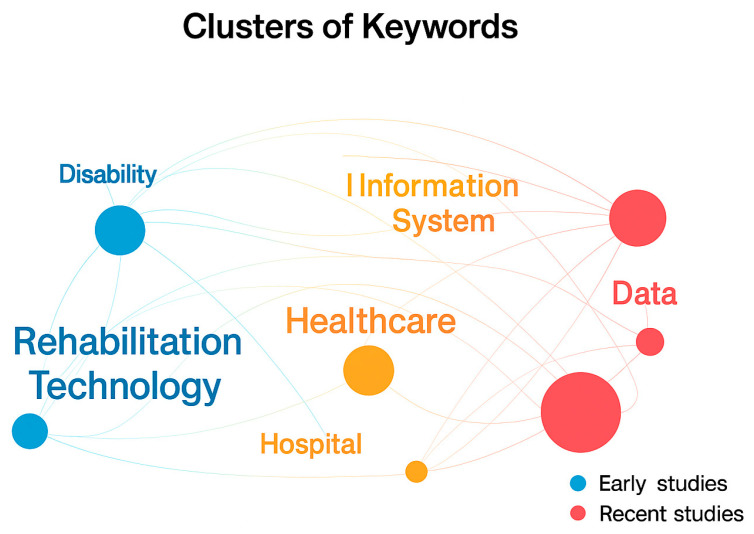
Overlay visualization of thematic evolution and collaboration networks in assistive technology research (2014–2024). Based on VOSviewer.

Figure 13 explains the following:

- Clusters of Keywords: The circles (nodes) represent key research terms such as “*Assistive Technology*,” “*Rehabilitation Technology*,” “*Healthcare*,” “*Information System*,” and “*Hospital.*” Each cluster reflects a thematic area—for example, “*Rehabilitation Technology*” (blue cluster) links to disability and mobility studies, while “*Healthcare*” and “*Information System*” (orange–red clusters) relate to digital health and data management.
- Connections (links): The lines connecting the nodes represent co-occurrence relationships, meaning that the connected keywords frequently appear together in publications, indicating conceptual or disciplinary overlap.
- Color Gradients: The color transition from blue to red represents publication chronology—blue for earlier studies and red for more recent ones. This shows thematic evolution, where research has shifted from rehabilitation and mobility (blue cluster) toward data-driven healthcare integration and information systems (red cluster).
- Node Size and Intensity: Larger or darker nodes indicate higher research activity or citation frequency, meaning those themes are more central to the field.

## 4. Discussion

Our first discussion focused on interpreting the following key observations:

1. *Rise of Publications After 2020:* The sharp increase in publications after 2020 corresponds to two global developments. First, the COVID-19 pandemic accelerated digital transformation and adoption of remote healthcare technologies, which in turn spurred research into assistive tools, telemedicine, and patient monitoring [1]. Second, international funding initiatives, such as the WHO Global Report on Assistive Technology (2022) and the AT2030 program, encouraged cross-disciplinary collaborations between computer science and health sciences to address care accessibility and resilience. These global health and policy shifts have led to a surge in research output over the past five years.
2. *Dominance of Computer Science over Medicine:* The predominance of computer science publications reflects the technology-driven evolution of assistive devices. Many innovations—such as wearable sensors, AI-driven monitoring systems, and machine-learning-based diagnostics—originate from engineering and computing fields rather than clinical medicine [7,13]. Medical journals often emphasize clinical trials and patient outcomes, whereas technological feasibility studies, prototype development, and algorithmic modeling are typically published in computer science venues. This disciplinary imbalance helps explain why technical papers outnumber clinical ones.
3. *Weak Keyword Linkage Between* “*Assistive Technology*” *and* “*Hospitals*”: The relatively low co-occurrence of the “assistive technology” with “hospital” keywords indicates that much of the AT literature remains focused on home care, rehabilitation, or community-based support rather than hospital-based clinical implementation. This suggests a fragmentation between technological development and clinical application. This limited overlap underscores the need for stronger interdisciplinary collaboration between engineers, healthcare practitioners, and policymakers to translate research prototypes into hospital-ready systems [5].
4. *Prevalence of Terms Such as* “*Rehabilitation Technology*” *and* “*Therapeutic Devices*”: These keywords appear more frequently because assistive technologies have been historically associated with physical rehabilitation, prosthetics, and therapy—areas with well-established research and clinical relevance. By contrast, the term “hospital assistive technology” is relatively new and inconsistently used in academic indexing. As the field broadens to include robotics, AI, and digital therapeutics, older terminologies such as *rehabilitation* and *therapeutic* remain dominant in keyword metadata [2]. This pattern reflects both linguistic inertia and established publication conventions across medical and engineering disciplines.
5. *Country and Institutional Dominance:* The bibliometric results show strong representation from the United States, Western Europe, and high-income Asian economies. This can be attributed to several systemic factors, including the following:
  ○ Policy and Funding: Governments in these regions actively support health-technology innovation through programs such as the EU Horizon framework and the U.S. National Science Foundation’s Smart Health initiative.
  ○ Technological Capacity: Advanced R&D ecosystems and university–industry partnerships foster innovation in robotics, IoT, and AI-based medical devices [17].
  ○ Publication Infrastructure: Researchers in these countries have greater access to indexed journals and English-language publication channels, contributing to higher visibility and citation strength. In contrast, low and middle-income countries face resource constraints, limited research funding, and infrastructural barriers that limit their presence in indexed AT research, despite substantial healthcare needs [31].

Taken together, these interpretations strengthen the discussion by connecting bibliometric outcomes to real-world structural, technological, and policy contexts. The revised section highlights that the dominance of certain countries, disciplines, and keywords is not random but reflects systemic differences in innovation capacity, policy incentives, and disciplinary focus—insights that are essential for shaping equitable and interdisciplinary growth in assistive technology research.

Interpreting Bibliometric Clusters and Co-Word Patterns:

This section interprets bibliometric clusters not merely as statistical groupings but as indicators of evolving technological maturity and the growing cross-disciplinary alignment between computer science, medicine, and healthcare management.

*Clusters Representing Stages of Technology Adoption:* The co-occurrence analysis shows that keywords such as “AI,” “machine learning,” and “IoT” cluster around “assistive technology” and “patient monitoring,” corresponding to the innovation and early adoption stages in Diffusion of Innovations Theory. In contrast, keywords like “*rehabilitation*,” “*therapy*,” and “*patient outcomes*” reflect the *implementation and impact evaluation* stages, where technologies have transitioned into practical healthcare settings and are evaluated for clinical efficacy.

*Clusters Reflecting Types of Assistive Systems:* The keyword network further differentiates between clusters focused on *physical rehabilitation systems* (e.g., “*mobility aids*,” “*prosthetics*,” “*rehabilitation devices*”) and those linked to *digital or cognitive assistive systems* (e.g., “*AI*,” “*wearable sensors*,” “*telehealth*,” “*mHealth*”). This pattern aligns with the *Human–Technology Interaction (HTI) and Socio-Technical Systems Theory*, which emphasize the co-evolution of technology design and clinical practice. The physical systems cluster represents traditional assistive approaches emphasizing device functionality, while the digital cluster illustrates the recent shift toward smart, data-driven, and context-aware assistive environments.

*Transition Toward Interdisciplinary Convergence:* The limited linkage between “*assistive technology*” and “*hospital*” suggests that the field is still in the early stage of interdisciplinary convergence, where engineering research is gradually being integrated into medical and clinical frameworks. This supports findings from *Translational Research Models*, which posit that technological innovation often precedes clinical assimilation and regulatory adaptation.

With the search term “assistive technologies in hospitals,” we conducted research on the 10 most cited articles in the Scopus database.

The reviewed articles underscore the transformative potential of assistive technologies (ATs) in healthcare, particularly in improving the quality of life for various patient groups and enhancing healthcare delivery. They demonstrate that assistive technologies can significantly enhance healthcare practices by improving patient care, streamlining clinical processes, and reducing costs. From elderly care and rehabilitation to global standards and custom fabrication, AT has proven to be a vital component in advancing healthcare delivery and improving patient outcomes across diverse settings. Table 9 provides an interesting data related to the papers on assistive technologies with the highest citations, as identified in the Scopus database.

Table 10 provides an explanation about how the top 10 articles have identified the impact of assistive technologies on healthcare practices.

Table 11 identifies how these top 10 articles have impacted the research landscape on assistive technologies.

The research discussed above provides valuable insights that may increasingly influence and shape future research in assistive technologies (ATs) in several significant ways.

Future studies in assistive technology (AT) are likely to build upon the findings and methods presented in these works. First, Article 1’s focus on systematic reviews and real-world evidence can help direct future research toward high-impact areas like mobility, social connectivity, and medication management. This ensures that new technologies directly address the core needs of target populations. Insights from Article 2 on technology acceptance among healthcare professionals can guide the design of more intuitive and efficient technologies, improving adoption and clinical integration. The call for international standards in Article 3 highlights the need for global collaboration and the development of universal guidelines, which can ensure consistent quality and accessibility of AT worldwide. Integrating advanced data management systems, as described in Article 4, can revolutionize how AT interacts with patient data, enabling more personalized and effective interventions. Article 5’s focus on custom fabrication highlights the growing potential for highly personalized AT solutions. This encourages more research into flexible, adaptable manufacturing technologies that can be made to fit each person’s needs. The innovative control interfaces explored in Article 6, such as hybrid BCI systems, open new possibilities for enhancing usability and accessibility for individuals with severe disabilities. Article 7’s example of using wearable tech like Google Glass in medical procedures points toward further research on augmented reality and related innovations that enhance procedural safety and precision. Article 8’s study on BCI systems for ALS patients emphasizes the importance of inclusive technologies that address diverse levels of impairment.

Article 9 illustrates how collaborative care models—especially for underserved populations—can integrate AT to enhance outcomes and reduce disparities. Lastly, the impact of home health nursing on pediatric care in Article 10 suggests that research should explore AT solutions that support home healthcare and long-term health management. Future research should also explore emerging technologies like AI, machine learning, and IoT to develop smarter, more adaptive AT solutions. It is also important to look into the social and economic factors that affect the use and effects of AT and to run large-scale clinical trials to prove the usefulness of new technologies. Collaborations between technologists, healthcare providers, and policymakers can drive innovation and ensure that AT solutions are both cutting-edge and widely accessible, ultimately enhancing the quality of life for diverse patient populations.

In addition to the well-known studies already discussed, other research also sheds light on emerging trends in assistive technologies. For instance, in pediatric healthcare, telehealth-based care teams have proven effective in providing ongoing, coordinated support for children living with chronic and complex conditions, helping to reduce hospital visits while ensuring that families receive timely advice and intervention [33]. Similarly, assistive technologies and telecare have enabled older adults with dementia to live independently for longer, reducing caregiver burden and delaying the transition to residential care [34]. When combined, these advancements suggest a future where telehealth and assistive devices will increasingly collaborate, providing remote monitoring, customized support, and seamless integration with smart home systems to enhance accessibility and ease of use. This approach supports medical needs while preserving independence, safety, and social connection among vulnerable users.

Issues in Bibliometric Analysis:

Several factors may explain why seemingly obvious keywords yield low search results in the bibliometric analysis (Table 4 and Table 5) in bibliometric analysis, as follows:

1. Synonyms and Variants: Different authors might use synonyms or variant terms for the same concept. For instance, “global warming” might be referred to as “climate change,” “environmental change,” or other related terms.
2. Field-Specific Jargon: Different academic fields might use specific jargon that is not obvious outside of that field. A keyword familiar in one field may be uncommon in another.
3. Keyword Specificity: Highly specific keywords or phrases may yield fewer results compared to broader terms. For example, “quantum entanglement in biological systems” is more specific and likely less common than “quantum entanglement.”
4. Temporal Factors: Some keywords may not have been commonly used during certain periods. If a term is relatively new or has only recently gained popularity, there might be fewer records available.
5. Database Coverage: Different databases have varying levels of coverage in terms of journals, conference papers, and other types of publications. A keyword might yield low search records in one database but higher records in another with more comprehensive or specialized coverage.
6. Indexing and Metadata Issues: Not all articles or publications might be properly indexed or tagged with the relevant keywords. This can result in lower visibility for certain terms, even if they are discussed in the literature.
7. Language Barriers: Research published in languages other than English might not use the same keywords or might not be indexed in databases predominantly containing English-language publications.
8. Publication Bias: Certain topics might be underrepresented in the literature due to publication bias, where only certain types of studies or results are published.

By considering these factors, researchers can better understand why certain keywords might not yield extensive search records and can adjust their search strategies accordingly.

Further, we need to be aware of several factors that can lead to very low citation counts and link strength (Table 7) in bibliometric analysis, as follows:

1. Recent Publications: Newly published articles have had less time to accumulate citations compared to older publications.
2. Niche Topics: Research on highly specialized or niche topics may have a smaller audience, resulting in fewer citations.
3. Journals With Low Impact Factors: Articles released in journals with lower impact factors may not have as many citations because fewer people read these journals.
4. Limited Accessibility: Articles that appear in journals that are hard for many people to read (for example, journals with paywalls or that are not well known) may be mentioned less often.
5. Poor Visibility: If major databases do not properly index or include research works, they may not receive as many citations.
6. Language Barriers: Articles written in languages other than English may not reach as many people, especially if most study articles are written in English.
7. Research Quality: Citations for articles with results deemed less important or of lower quality may be less frequent.
8. High rates of self-citation can sometimes obscure a paper’s true impact. Conversely, papers with few self-citations may also show lower overall citation counts.
9. Disciplinary Practices: The way people in different fields cite sources is very different. Different fields might have different guidelines or cite earlier work differently or with different priorities.
10. Lack of Awareness: Some studies may not have as many citations if researchers do not know about them. This could be because they have not been promoted or shared enough.
11. Lack of or Negative Results: Studies that report negative or null results usually have fewer links than studies that report positive or new results.
12. Obsolescence: Older studies may be seen as irrelevant or out of date, which causes fewer citations over time.

Researchers can better understand bibliometric data and come up with ways to make their work more visible and have a bigger effect if they understand these factors.

Future Research on Assistive Technologies:

To strengthen the future of assistive technology (AT), research should prioritize several key areas, building on existing knowledge.

User-Centered Design and Usability: Ensure AT solutions meet user requirements, such as for healthcare workers and patients, by focusing on user-centered design. Systems should be intuitive and accessible, helping identify factors that influence user acceptance and sustained use.

Personalization and Customization: Make user-specific, personalized, and flexible AT solutions. This includes improvements in rapid prototyping and fabrication methods that could help make customizable AT products. For example, we can explore how assistive technology (AT) can integrate with clinical data stores and natural language processing tools to enhance advanced data management. This can make assistive technology (AT) easier to use by enabling more personalized, data-driven actions. A recent citation in support of the above AT is *User*-*Centered Insights into Assistive Navigation Technologies for Individuals with Visual Impairment* [35]. This April 2025 mixed-methods study engaged 19 individuals with visual impairment through surveys and virtual workshops to explore their needs, preferences, and barriers in adopting navigation technologies.

Global Standards and Accessibility: We need to promote international AT standards to ensure global quality and accessibility. Researchers should work toward developing global standards that ensure equitable access to high-quality assistive technologies for all individuals with disabilities. A recent citation relevant to this topic is the scoping review titled “Global Availability of Guidelines Related to Assistive Technology” by [36]. This scoping review mapped 291 records (2008–March 2024), identifying only 24 globally available guidelines covering AT, mostly from high-income countries, and focused on mobility, vision, hearing, and self-care products.

New Technologies: AT could work much more efficiently with new technologies like artificial intelligence (AI), machine learning, and the Internet of Things (IoT). We need more studies on mixed control interfaces, such as brain–computer interfaces (BCIs) that use both EMG channels and BCIs. By using residual muscle activity, these interfaces can make it easier for people with serious motor disabilities to use them. Wearable and Augmented Reality Technologies: Hospitals can leverage wearable and augmented reality (AR) technologies to improve procedural safety and precision. More research is needed on how these technologies can assist healthcare workers in their daily tasks. A recent study on this topic is “Hybrid Brain-Machine Interface: Integrating EEG and EMG for Reduced Physical Demand” [37]. This study demonstrates a hybrid EEG–EMG brain–machine interface (BMI) that dynamically switches between steady-state visually evoked potential (EEG-SSVEP) and facial EMG control. EEG and EMG are both methods used to measure electrical activity in the body, and they are commonly used in assistive technology (AT) and brain–computer interface (BCI) research.

Collaborative Care Models: Greater investment is needed in developing collaborative care models that integrate AT into comprehensive care frameworks. This type of intervention is especially important for underserved groups, thereby minimizing health disparities and making it easier for people to receive integrated care. An intriguing study in this context is *Towards Co*-*Design in Delivering Assistive Technology Interventions* [38]. This study introduces an AT collaboration tool that defines roles for consumers, allied health practitioners (AHPs), support networks, and support workforce across service steps from assessment to follow-up.

Healthcare for Children and at Home: Research should examine how AT can support long-term health management, reduce hospitalizations, and improve outcomes for children with complex medical needs. A U.S. telehealth complex care model serving medically complex children showed reduced healthcare utilization even though participants had higher risk profiles. The model emphasized proactive telehealth follow-up, care coordination, and early detection of care gaps.

Socioeconomic Factors and Policy Research: Policymakers and researchers should collaborate to make AT solutions affordable and accessible across all income levels. *Assistive Products Market Report 2025*, see [31], is an important source in this context. This report highlighted that over 1 billion people worldwide still lack access to essential assistive products, primarily in low- and middle-income countries (LMICs). This report further highlights critical barriers: fragmented policies, inadequate funding, and high out-of-pocket expenses—users often pay up to six times the ex-factory cost, with 65–95% of those in need remaining unserved in LMICs. The WHO Global Report [19] and AT2030 program emphasize universal coverage, public subsidies, and equitable AT access [39].

Large-Scale Clinical Trials and Longitudinal Studies: To prove that AT solutions work and have long-term effects, large-scale clinical trials and longitudinal studies are being carried out. Such studies are vital, as they provide the robust evidence needed to support broader adoption of AT in healthcare.

Large-scale trials with long-term follow-up (e.g., ATTILA, WSD) are essential for evaluating the real-world effectiveness and cost-effectiveness of AT interventions. With the ATTILA Randomized Controlled Trial (UK) in 2021, a pragmatic RCT involving 495 people with dementia was conducted to assess whether assistive technology (AT) and telecare extend safe independent living at home and prove cost-effective. Over a 2-year follow-up, this trial found no significant AT benefit, underscoring the limitations of real-world delivery.

Interdisciplinary Collaboration: Technologists, healthcare providers, lawmakers, and end users need to work together across disciplines. Such interdisciplinary collaboration fosters innovation, enhances AT effectiveness, and accelerates real-world application of research findings. The study [40] emphasizes the collaborative nature of interdisciplinary special education and how assistive technologies can enhance collaboration among educators, therapists, and specialists to improve the learning outcomes of students with disabilities.

Current analysis highlights four major domains where targeted action is needed for the future, as follows:

1. Integration Challenges: Many hospitals still rely on outdated information systems that are incompatible with modern assistive technologies like wearables, IoT-based monitors, and AI-enabled diagnostics. Multiple studies highlight the technical and organizational challenges of incorporating these systems into existing hospital workflows [7,13]. These findings encourage healthcare providers to prioritize investments in digital infrastructure and interoperable platforms that support seamless clinical integration.
2. Data Privacy and Security: The issue of patient data protection and ethical use of digital health records remains underexplored in many studies, despite growing adoption of data-driven assistive systems. Earlier studies [7,19] echo concerns about data security in real-time monitoring and cloud analytics, underscoring the need for stronger privacy frameworks and standardized cybersecurity policies for AT deployment.
3. Interoperability: Bibliometric patterns show that various assistive technologies and hospital systems often operate in silos, causing fragmented data management and limited interoperability. As highlighted by [5,17], developing global standards for interoperability and communication protocols between assistive devices and hospital information systems is essential for large-scale adoption. This aligns with the WHO’s recommendation for international cooperation in developing unified standards for assistive product design and deployment.
4. Cost-Effectiveness: The review revealed a shortage of longitudinal and economic studies evaluating the long-term cost-effectiveness of assistive technologies in clinical settings. Studies such as [4,12] underline that cost–benefit evaluation remains an important but under-researched factor in healthcare decision-making. Addressing this gap will enable policymakers to design sustainable funding models and encourage private-sector participation through incentives and public–private partnerships.

By identifying and explaining these gaps, this study contributes practical insights that can support evidence-based policy formulation, targeted investment, and strategic planning. The findings thus provide a framework for both public and private healthcare institutions to implement assistive technologies in a way that is integrated, secure, interoperable, and economically sustainable.

Major Potential Initiatives and Research Directions for the Future:

1. Integrating AI and Assistive Technologies in Healthcare

Giansanti in [41] discusses the transformative potential of integrating artificial intelligence (AI) into assistive technologies, aiming to enhance autonomy and quality of life for individuals with disabilities and aging populations. The review identifies prevailing themes, opportunities, challenges, and recommendations regarding the integration of AI in assistive technologies. AI is becoming essential for advancing mobility, diagnostics, and cognitive support, providing more personalized and adaptive user experiences. Incorporating AI into traditional assistive technologies like smart wheelchairs and exoskeletons boosts their responsiveness and intuitiveness. Additionally, AI-based tools are fostering inclusion for children with autism spectrum disorders by supporting social interaction and cognitive growth. The review concludes by outlining both emerging opportunities and persistent challenges in assistive technology research.

2. Assistive Technologies in Healthcare: Utilization and Impact

Sommer in [42] broadens the scope of assistive technology to include tech that assists healthcare workers, aiming to enhance efficiency, autonomy, and the ability to deliver high-quality care. The study stresses that interdisciplinary collaboration is key to creating assistive technologies that support both healthcare professionals and patients.

3. How Cross-Disciplinary Collaboration is Transforming Assistive Tech and Student Learning

Horency in [43] highlights the role of cross-disciplinary collaboration in transforming assistive technology and student learning. At George Washington University, occupational therapy doctoral candidates and biomedical engineering students collaborated to develop a glove prototype that detects and translates sign language into on-screen text. This initiative showcases how cross-disciplinary collaboration can yield innovative assistive technology solutions.

4. Fostering Inclusion: A Regional Initiative Uniting Communities to Co-Design Assistive Technologies

In [44], it is described as a regional initiative that brought together people with disabilities, students, researchers, and associations to co-design assistive technologies. The initiative sought to address challenges of affordability and suitability in AT while promoting social awareness and acceptance of disability. Participants valued the co-design model, which underscored the role of collaboration, continuity, and public engagement in AT development.

5. Assistive Technologies in Interdisciplinary Special Education

Study [40] provides a comprehensive review of current practices utilizing assistive technologies to enhance collaboration in interdisciplinary special education. The study explores the significance of assistive technologies in supporting diverse learners with disabilities and assesses their effectiveness in promoting inclusive education. It highlights the collaborative nature of interdisciplinary special education and how assistive technologies can enhance collaboration among educators, therapists, and specialists.

Challenges in Assistive Technology Plan Implementation in Hospitals:

Integration with Existing Systems: Integrating legacy hospital systems and practices with modern assistive technologies remains challenging and often incompatible. Linking AT systems with electronic health records (EHRs), patient management tools, and other infrastructure can be technically complex.

User Training and Adaptation: While evidence confirms the benefits of AT, many studies overlook the critical need for healthcare professional training and ongoing support. Large hospitals often resist system changes, making it essential for administrators to ensure that staff are adequately trained and confident in using AT.

Cost and Funding: AT may involve high procurement, setup, and running costs, especially in places with few resources. Few studies have examined sustainable funding models or long-term cost-benefit analyses to guide investment decisions in AT.

Customization and Scalability: Robust operational strategies are needed to ensure AT solutions can be customized and adapted for diverse patient populations and clinical settings. Research rarely examines how these methods can be scaled or adapted to new contexts without compromising efficiency or effectiveness.

Interoperability issues: The lack of standardization and compatibility among AT systems hinders usability and integration. Interoperable options that can work together without major hiccups in a hospital’s ecosystem are yet to be fully researched.

Concerns about security and privacy: Data security and patient privacy become more important than ever with the rapid growth of digital and connected AT devices. Further research is needed to develop robust security protocols and privacy safeguards for patient data protection.

Patient Acceptance and Engagement: Healthcare workers receive a lot of attention, but patients also need to be able to accept and use AT systems to function effectively. Researchers should explore strategies to build patient trust and engagement with AT tools.

Regulatory and Compliance Issues: Ensuring consistent regulatory standards and compliance across medical equipment remains a major challenge. There is inadequate research on the legal issues and methods for approving and using new AT solutions in hospitals.

Impact and Effectiveness in the Long Term: Most studies emphasize short-term AT benefits, with limited research on its long-term impact on patient outcomes and hospital performance. Longitudinal studies are needed to determine how beneficial AT is over time.

Interdisciplinary Collaboration: We need people from different fields to work together for AT to be used effectively. This includes healthcare providers, technologists, administrators, and lawmakers. Researchers have yet to determine effective strategies for initiating and sustaining interdisciplinary collaboration.

Change Management: Implementing AT often requires significant operational and procedural changes within hospitals. There is limited research on effective change management strategies to facilitate smooth AT adoption with minimal disruption.

Ethical Considerations: Ethical aspects of using AT remain under-researched, such as making sure everyone has equal access and avoiding possible biases in the use of technology. Stakeholders must ensure that AT is implemented equitably and inclusively, avoiding bias or unequal access.

**Figure 14:**
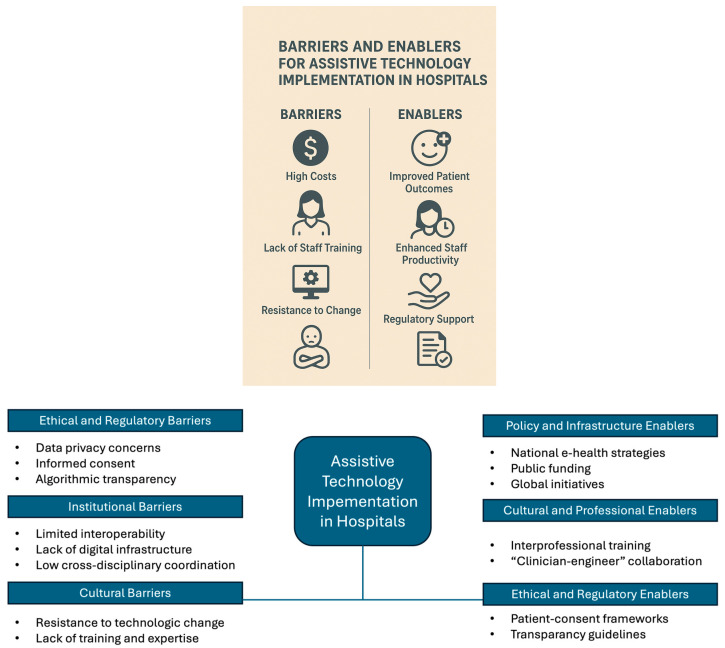
Barriers and enablers for assistive technology implementation in hospitals.

Ethical and Policy Frameworks for Assistive Technology Integration:

Although ethical and policy issues have been recognized in previous literature, this study goes beyond listing them and integrates these dimensions into a conceptual synthesis of barriers and enablers influencing assistive technology (AT) implementation in hospital care. Figure 14 has identified the barriers and enablers for assistive technology implementation in hospitals.

The findings reveal that ethical and regulatory barriers—including concerns about data privacy, informed consent, and algorithmic transparency—remain insufficiently addressed in current research and often delay institutional adoption [13,19]. At the organizational level, institutional barriers such as limited interoperability, lack of digital infrastructure, and low cross-disciplinary coordination further constrain integration. Conversely, policy and infrastructural enablers—notably national e-health strategies, public funding, and the WHO’s global initiatives on accessible technologies—serve as catalysts for scaling implementation.

Anchoring these findings within established theoretical models provides greater clarity in understanding the translational gap between technological innovation and clinical adoption. Drawing on the Technology Acceptance Model (TAM) and the Diffusion of Innovations (DOI) framework, this study interprets the identified barriers and enablers (Figure 13) as interconnected determinants influencing assistive technology (AT) adoption in hospital environments.

From a TAM perspective, ethical and regulatory issues—such as data privacy, informed consent, and algorithmic transparency—directly affect user trust, perceived usefulness, and perceived ease of use, which are central to technology acceptance. Institutional barriers, including limited interoperability and inadequate digital infrastructure, further constrain perceived system efficiency and reliability.

Through the DOI lens, policy and infrastructural enablers such as national e-health strategies, public funding, and the WHO’s global initiatives act as facilitators of diffusion, enhancing the compatibility, observability, and trialability of assistive technologies within healthcare systems. Moreover, cultural and professional enablers, including interprofessional training and clinician-engineer collaboration, contribute to adoption readiness by promoting ethical alignment and cross-disciplinary communication.

Overall, situating the findings within these theoretical frameworks strengthens the interpretive coherence of the study. It demonstrates that the successful integration of assistive technologies in hospitals depends not only on technological innovation but also on the alignment of ethical governance, institutional capacity, and policy support mechanisms that collectively drive sustained adoption and impact.

Finally, cultural and professional enablers, such as interprofessional training and clinician–engineer collaborations, strengthen adoption readiness and ensure ethical alignment in real-world applications. By organizing these factors into a coherent framework, the study demonstrates that AT adoption in hospital environments depends not only on technological advancement but also on the balance between ethical governance, institutional capacity, and supportive policy ecosystems.

Future studies should focus on strengthening health infrastructure through clearer frameworks for implementing assistive technologies that both enhance patient outcomes and improve hospital efficiency.

## 5. Conclusions

Recent bibliometric analyses underscore key emerging trends in assistive technology, including the increasing integration of augmented reality (AR) to enhance accessibility for individuals with disabilities. As [45] highlight, AR’s ability to provide real-time, context-aware support represents a significant shift toward more immersive and personalized AT solutions, signaling promising directions for future research and development in this field.

Recent studies increasingly agree that these emerging technologies could fundamentally transform patient care and overall hospital operations [13]. Within the research domain, systematic reviews, empirical studies, and position papers highlight several key areas where AT can have a significant impact. For example, these technologies can enhance mobility, social connection, and rehabilitation outcomes, offering increasingly personalized and beneficial healthcare experiences. However, researchers will need to attend to specific problems before these technologies can be used successfully in both hospitals and homes.

Hospital Settings: Setting up AT in a hospital and figuring out how to connect it to the current systems and workflows is challenging due to complicated legacy infrastructure. Therefore, establishing a structured training infrastructure is essential to ensure effective adoption and adaptation of new technologies by healthcare staff. Additionally, hospital administrations should explore sustainable funding models to prevent high procurement, installation, and maintenance costs from becoming barriers. Compatibility challenges must also be addressed to enable seamless integration between diverse AT systems and hospital technologies. Moreover, robust data privacy and security measures must be maintained to safeguard patient trust. Regulatory and compliance landscapes are even more complicated to navigate and require a lot of study and planning. Long-term studies of effectiveness and effects are needed to prove that AT has long-lasting benefits. Cross-sector collaboration among technologists, healthcare professionals, administrators, and policymakers is essential to drive innovation and successful implementation. Lastly, good change management strategies are needed to make sure that transitions go smoothly and that hospital processes are interrupted as little as possible.

Home Settings: Although distinct, challenges in implementing AT within home settings are equally critical. User-friendly design is essential, as patients and caregivers may lack the technical expertise available in clinical environments. The main goal of research should be to make AT systems that are simple and easy to use. Home-based AT should also be adaptable and scalable to meet diverse user needs and living conditions. Cost remains a significant barrier, particularly for individuals and families. This is why we need to investigate more affordable AT choices and funding assistance programs. Another challenge involves ensuring synchronization between home-based AT devices and broader healthcare systems. Effective use of home-based AT depends on patient willingness, confidence, and perceived safety. This requires ongoing help and education. Regulatory expectations and quality standards differ significantly between home and hospital environments. Longitudinal research is needed to assess the long-term effects of AT on health outcomes and quality of life in home settings. Equitable use of AT must also be ensured, addressing ethical concerns such as equal access and bias-free implementation.

As research moves forward, we need more in-depth analysis of how to find new solutions that will make it easier for people to use assistive tools at home and in hospitals. Only by tackling these complex problems can we create a healthcare system that is more flexible, quick, and aware of the different needs of its patients. This will eventually lead to better health results and quality of life for everyone.

## Figures and Tables

**Table 1:** Inclusion criteria.

Publication Type	Language	Time Frame	Study Focus
● Peer-reviewed journal articles ● Conference proceedings ● Systematic reviews and meta-analyses ● Research studies (qualitative, quantitative, and mixed-method)	English language publications	Studies published within the last 20 years (2004–2024) to capture recent developments and trends.	● Research specifically on assistive technologies used in hospitals. ● Studies evaluating the effectiveness of these technologies in patient profiling (e.g., diagnostic tools, patient monitoring systems) and treatment (e.g., rehabilitation devices, robotic surgery). ● Papers discussing the impact of assistive technologies on patient outcomes, healthcare efficiency, or clinical workflows.
Population		Geographical Scope	
● Studies involving hospital patients of any age group. ● Research involving healthcare professionals who use assistive technologies in hospital settings.		No geographical restrictions to capture a global perspective.	

**Table 2:** Exclusion criteria.

Publication Type	Language	Time Frame	Study Focus
● Non-peer-reviewed articles (e.g., opinion pieces, editorials, letters to the editor). ● Abstracts without full-text availability. ● Theses and dissertations.	Non-English language publications.	Studies published before 2004.	● Research focusing on assistive technologies used outside hospital settings (e.g., home care, outpatient clinics). ● Studies not directly evaluating the effectiveness of assistive technologies (e.g., purely theoretical papers, technology development without evaluation). ● Papers focusing solely on non-medical assistive technologies (e.g., educational technologies for disabled students).
Population		Geographical Scope	
● Studies involving non-hospitalized patients exclusively. ● Research involving assistive technologies for caregivers or family members without direct application to patient care in hospitals.		None, to allow for a comprehensive global analysis.	

**Table 5:** Search results using phrases that should be searched together for meaningful results.

Search String: Limited to Medicine, Health Professions, Engineering, Social Sciences, and Computer Science Limited to Articles, Conference Papers, Reviews, and Book Chapters	Number of Search Results (2014–2024)
“Assistive technologies” AND “Hospital” AND “Patient outcomes”	5
“Assistive technologies” AND “Hospital” AND “Clinical outcomes”	4
“Assistive technologies” AND “Hospital” AND “Healthcare efficiency”	0
“Assistive technologies” AND “Hospital” AND “Clinical workflow”	1
“Assistive technologies” AND “Hospital” AND “Treatment efficacy”	0
“Assistive technologies” AND “Hospital” AND “Patient care”	18

**Table 6:** Search results using phrases that falls under the broader topic titled “Assistive Technologies in Hospital Settings”.

Search String: Limited to Medicine, Engineering, and Computer Science Limited to Articles, Conference Papers, Reviews, and Book Chapters	Number of Search Results (2014–2024)
Medical assistive devices	20
Healthcare assistive technology	7
Rehabilitation technology	533
Adaptive medical equipment	0
Health support technology	3
Patient assistive systems	3
Assistive medical solutions	0
Therapeutic technology	182
Clinical assistive technology	1
Mobility aids in healthcare	0

**Table 7:** Search results using phrases that should be searched for as realistic alternatives to “Assistive Technologies in Hospital Settings”.

Search String: Limited to Medicine, Engineering, and Computer Science Limited to Articles, Conference Papers, Reviews, and Book Chapters	Number of Search Results (2014–2024)
“assistive technology” for “hospitals”	251
“assistive technology” in “hospitals”	245
“assistive technology” AND/FOR “treatment”	467

**Table 8:** The total number of occurrences of the 12 most important keywords with total link strength.

Keyword	Occurrences	Links	Total Link Strength
Human	6	11	38
Male	5	11	36
Adult	4	10	32
Assistive Technology	7	11	32
Female	4	10	32
Humans	3	10	25
Self-help Devices	3	10	25
Aged	3	10	24
Cerebrovascular Accident	3	10	24
Middle Aged	3	10	24
Article	4	11	21
Hospitals	3	4	5

**Table 9:** Articles with the highest citations (top 10).

Article		Citation Count
1	Investigating the effectiveness of technologies applied to assist seniors: A systematic literature review [4]	220
2	What factors determine therapists’ acceptance of new technologies for rehabilitation-a study using the Unified Theory of Acceptance and Use of Technology (UTAUT) [6]	132
3	Assistive technology provision: towards an international framework for assuring availability and accessibility of affordable, high-quality assistive technology [5]	121
4	A clinician-friendly data warehouse oriented toward narrative reports: Dr. Warehouse [8]	93
5	[9]	56
6	Hybrid P300-based brain-computer interface to improve usability for people with severe motor disability: Electromyographic signals for error correction during a spelling task [10]	53
7	Ultrasound-guided central venous access using Google glass [11]	46
8	Assistive device with conventional, alternative, and brain-computer interface inputs to enhance interaction with the environment for people with amyotrophic lateral sclerosis: A feasibility and usability study [32]	44
9	Technology-facilitated depression care management among predominantly Latino diabetes patients within a public safety net care system: Comparative effectiveness trial design [11]	38
10	Home health nursing care and hospital use for medically complex children [12]	37

**Table 10:** Explanation of the top 10 articles—impact of assistive technologies on healthcare practices.

	Impact of Assistive Technologies on Healthcare Practices
1	Article 1 highlights a systematic review showing that AT can significantly improve seniors’ mobility, social connectedness, and medication management, reducing hospital readmissions. Key technologies include ICT, robotics, telemedicine, sensors, and medication management applications.
2	Article 2 examines the acceptance of rehabilitation technologies by therapists, emphasizing that performance expectancy is crucial for adoption. Therapists prioritize the benefits of technology over the effort required to use it and social pressures, with institutional support being essential for technology use.
3	Article 3 advocates for an international framework to standardize AT provision, aiming to improve accessibility and quality globally. Such standards could ensure that people with disabilities have better access to affordable and high-quality AT solutions.
4	Article 4 describes Dr. Warehouse^®^, a clinical data warehouse focusing on narrative reports. It supports translational research and clinical use, demonstrating high user satisfaction and extensive data utilization.
5	Article 5 explores clinician involvement in AT fabrication, finding that existing rapid prototyping processes do not align well with clinical practices. Recommendations include software solutions to integrate fabrication into client care, enhancing clinician ability to create tailored AT.
6	In Article 6, a hybrid BCI system for controlling AT is looked at. It is found to be more useful and has potential benefits for people with severe motor disabilities. This system integrates BCI and EMG channels to enhance communication and environmental interaction for such patients.
7	Article 7 investigates the use of Google Glass during ultrasound-guided procedures. By projecting images into the practitioner’s view, this technology may reduce unintentional hand movements, improving procedural accuracy and safety.
8	Article 8 tests an AT prototype for people with ALS and shows that a P300-based BCI can work as an input method without making the device less usable. This enables patients with varying degrees of muscular impairment to maintain communication and control their environment.
9	Article 9 focuses on a technology-facilitated model for collaborative depression care in Hispanic/Latino patients with diabetes. By integrating automated telephonic screening and a comprehensive care management registry, this model aims to reduce health disparities and improve care outcomes.
10	Article 10 demonstrates that home health nursing services reduce hospital readmissions, admissions, days in the hospital, and costs for children with medical complexity. This highlights the significant impact of HH care on pediatric health and resource utilization.

**Table 11:** Explanation of the top 10 articles—impact on the assistive technology research landscape.

	Impact on the Assistive Technology Research Landscape	Research Focus
1	Article 1 underscores the importance of systematic reviews to identify key areas where AT can benefit seniors, such as mobility, social connectedness, and medication management. This approach can guide researchers to focus on technologies that address the most pressing needs, ensuring that future studies build on a solid foundation of empirical evidence.	Evidence-based evaluation
2	Article 2 shows that understanding the factors that affect healthcare workers’ acceptance of AT is important. This can push researchers to make technologies that are easy for therapists and other healthcare professionals to use and meet their practical needs. This can lead to higher adoption rates and better usage in clinical settings.	Focus on user-centered design
3	Article 3 mentioned the importance of setting international guidelines for providing AT. This could ensure consistent quality and availability of AT around the world. This could also encourage researchers to work together across countries and help make universal rules ensuring everyone has equal access to AT.	Standardization and global accessibility
4	Article 4 shows the use of advanced data management tools, like Dr. Warehouse^®^, in clinical settings. This research shows that combining natural language processing and other data management tools could make AT more user-friendly and beneficial. This leads to better decision-making based on data and could potentially improve patients’ health.	Integration of advanced data management
5	Article 5 talks about the pros and cons of making unique ATs. An increasing body of work is now focused on making fabrication methods and tools that are adaptable and simple to use in hospitals. This can potentially create personalized AT solutions customized to each patient’s needs.	Customization and personalization
6	Article 6 talks about how hybrid control interfaces could help people who have severe motor disabilities. Such a technology could be used in assistive technology (AT) devices to make them more accessible.	Innovative control interfaces
7	Article 7 talks about how new technologies, such as Google Glass, can be useful in clinical processes. This allows us to further study wearable and augmented reality technologies that can make medical processes safer and more accurate, which could change the way we operate now.	New procedural technologies
8	Article 8 talks about how hybrid control interfaces could be used to help people with ALS, which encourages more study into AT solutions for people with severe disabilities. This allows researchers to create technologies that help with everyday activities, thereby providing more freedom and a better quality of life.	Broadening accessibility for severe disabilities
9	Article 9 talks about how technology can facilitate the functioning of joint care models, especially for neglected groups. In the future, researchers can work on making these models better and combining AT with full care management systems to make health results better and reduce differences.	Collaborative care models
10	Article 10 shows how home health care can help keep children with complicated medical needs out of the hospital. Based on these results, researchers can look into the role of AT in home healthcare and create tools that help with long-term health management and lower the number of hospital stays.	Impact on pediatric care

## Data Availability

No new data were created or analyzed in this study.
